# 

**Published:** 1888-08

**Authors:** 


					﻿; Vol. VIII.
AUGUST, 1888.
NO. &
THE
HOMEOPATHIC pHY£l(MjI,
A Monthly Journal of Homoeopathic Materia Medica
.and Clinical Medicine.
“ He is ci freeman whom truth makes free, and all are slaves beside.”
“Seek the Truth: come whence it%may, cost what it will.”
EDITED AND PUBLISHED BY
Edmund j. lelr, Mi d.,
AND
WALTER M. JAMES, M. D.
PHILADELPHIA:
No. 1123 8PRTTC1C STREET.
, *	TiOYSTXJOITs
ALFRED HEATH & CO., Sole Agents foe Great Britain,
114 Ebury Street, Eaton Square.
Yearly Subscription, $2.50, invariably in advance. Single numbers, 9S cts.
Entered at the Philadelphia Poet-Office as Second-Class Mall Matter.
				

